# Evidences of Polymorphism Associated with Circadian System and Risk of Pathologies: A Review of the Literature

**DOI:** 10.1155/2016/2746909

**Published:** 2016-05-22

**Authors:** F. J. Valenzuela, J. Vera, C. Venegas, S. Muñoz, S. Oyarce, K. Muñoz, C. Lagunas

**Affiliations:** ^1^Department of Basic Sciences, Universidad del Bío-Bío, Campus Fernando May, 378000 Chillán, Chile; ^2^Group of Biotechnological Sciences, Department of Basic Sciences, Universidad del Bío-Bío, Campus Fernando May, 378000 Chillán, Chile

## Abstract

The circadian system is a supraphysiological system that modulates different biological functions such as metabolism, sleep-wake, cellular proliferation, and body temperature. Different chronodisruptors have been identified, such as shift work, feeding time, long days, and stress. The environmental changes and our modern lifestyle can alter the circadian system and increase the risk of developing pathologies such as cancer, preeclampsia, diabetes, and mood disorder. This system is organized by transcriptional/tranductional feedback loops of clock genes* Clock*,* Bmal1*,* Per1–3,* and* Cry1-2*. How molecular components of the clock are able to influence the development of diseases and their risk relation with genetic components of polymorphism of clock genes is unknown. This research describes different genetic variations in the population and how these are associated with risk of cancer, metabolic diseases such as diabetes, obesity, and dyslipidemias, and also mood disorders such as depression, bipolar disease, excessive alcohol intake, and infertility. Finally, these findings will need to be implemented and evaluated at the level of genetic interaction and how the environment factors trigger the expression of these pathologies will be examined.

## 1. Introduction

The circadian system organizes the different biological functions in 24 h, such as sleep/activity, temperature [1], heart rate, glucose level, cortisol production [2], and oxidative stress [3]. In mammals, this system is organized by a central clock localized in the suprachiasmatic nucleus of the hypothalamus (SCN) and in a series of peripheral oscillators such as the liver, lung, adrenal gland, fibroblast cells, and others tissues [2, 4]. The peripheral oscillators are synchronized every day via nervous or humoral signals, and the most important humoral signal is the melatonin hormone, secreted by the pineal gland during the dark hours, and its impairment is associated with different disorders such as insomnia, cardiovascular disease, and cancer [5].

**Figure 1:**
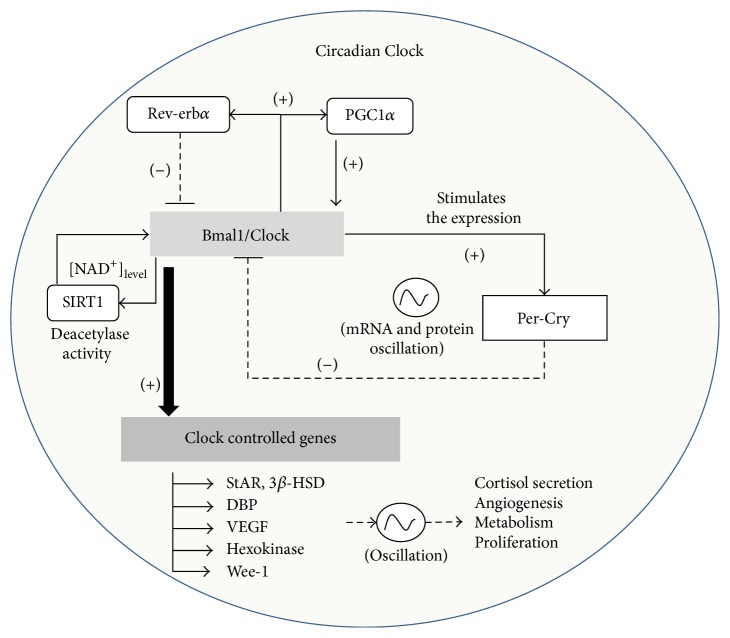
Circadian Clock. Positive regulation of clock genes* Bmal1* and* Clock* stimulates promoter of negative regulators* Per1–3*,* Cry 1-2,* and controlled clock genes StAR, 3*β*-HSD, DBP, VEGF, Hexokinase, and Wee-1. Moreover, the molecular clock regulates the deacetylase activity of SIRT1 by regulation of NAD^+^/NADH ratio.

The molecular clock is organized by transcriptional/translational feedback loop of clock genes named* Clock*,* Bmal1*,* Per1–3*, and* Cry1-2*. At the molecular level, the complex Clock-Bmal1 stimulates the expression of negative regulators* Per1–3* and* Cry1-2*, and their protein inhibits the effect of the heterodimer Clock/Bmal1 [6, 7]. Moreover, the molecular clock has different modulators which give fine tuning of output signals such as Rev-erb*α*, a negative regulator of BMAL-1 expression [7–9], SIRT1, a regulator of Clock-mediated acetylase activity [10, 11], and PGC1*α*, a stimulator of Bmal1 expression [12, 13] (see Figure 1). This system provides an output signal to genes such as Hexokinase [14], DBP [15–17], VEGF [18], steroidogenic enzymes StAR, 3*β*-HSD [19], and Wee-1 [20], giving a circadian oscillation of physiological functions such as metabolism [14], angiogenesis [18], cortisol production [2, 19], and cellular proliferation [20].

The molecular clock can be modified by environmental changes and our modern lifestyle, resulting in physiological alteration and risk of pathologies; for example, during the monkey's pregnancy, the light exposition during night hours induces a lower body temperature and absence of circadian rhythm of temperature in the newborn [1]. In humans, different reports showed that the alterations of the circadian system increased the risks of cancer [21–23], preeclampsia [24], diabetes [3, 25], and mood disorder [26]. Curiously, a large quantity of polymorphisms has been detected in clock genes, which can be influencing the development of diseases through different physiological systems. This review will primarily focus on the hypothesis which states that the variation of genetic components of the circadian system, similar to environmental changes, can affect several physiological systems and produce an elevation of the risk of developing a disease.

In recent years, there has been a significant increase in available information from polymorphic variations and epigenetic modification over clock genes and their risk of diseases.

The report of health from the United States (2014) showed about 10% of population have a poor health associated with pathologies such as obesity (35.5%), hypercholesterolemia (30%), diabetes (32%), and cancer (6.4%) [27]. Curiously, despite its low frequency in population, cancer is the second cause of death in the United States [27]. The above pathologies add the infertility, which affects about 10.9% of women in the United States [27] and 17% of women in other developed countries [28]. During short-stay in the hospital, mood disorder and psychosis are the principal causer of hospital stay during 2014 and represent the third cause of morbidity worldwide [29]. This present research reports the last single nucleotide polymorphism (SNP) associated with an elevated risk of developing principal pathologies worldwide such as mood disorder [30], infertility [31], cancer [32], metabolism and diabetes [33] and addictions [34], and a description of usefulness for a more detailed study in other pathologies.

## 2. Clock Genes and Metabolic Disorder

In mammals, the circadian system is a supraphysiological system that regulates biological functions every 24 h [35] such as glucose homeostasis [36], temperature [1], blood pressure, tone in the coronary artery, and heart rate [37]. The Bmal1/Clock complex target genes are related to the circadian expression of metabolic pathways as was observed by Hatanaka et al. [38] through high resolution genome-wide mapping in the liver of mice. The author detected a circadian expression of (I) glucose metabolism-related genes such as* Glut2*,* Por*,* Pck1,* and* Gys2* and (II) cholesterol metabolism-related genes such as* Cyp2a4*,* Cyp2a5*,* Cyp4a14*,* Cyp7a1*, and* Cyp2c55* [38]; and considering the very important role these systems have in metabolism, any alteration of them can have negative health effects.

Today there are several metabolic problems affecting the population, such as obesity, hyperglycemia, dyslipidemia, and hypertension [39]. The cooccurrence of three or more metabolic disorders, including obesity, is defined as “metabolic syndrome” [39], showing a prevalence in the United States during 2009-2010 of about 22% of adults (21.46–26.15) [40]. The insulin resistance or type 2 diabetes is one of the most important metabolic diseases currently affecting about 366 million people [41], causing more than 3.8 million deaths [42], and showing a rise in the number of affected people as observed in the United States population during 1999–2010 [40]. Several factors could be increasing the risk of death by diabetes, such as dyslipidemia, hypertension, and obesity [43], all of which are driving to an elevated risk of cardiovascular disease. A variant of this pathology can also develop during the pregnancy (gestational diabetes mellitus) and is characterized by glucose intolerance [44] in the mother and adverse consequences for her offspring, such as an increase in blood pressure, body mass index (BMI), and body fat and a decrease in HDL levels [43]. At the molecular level, both diseases have in common the impairment of signal transduction of insulin receptors such as PI3Kinase/Akt and Ras/MAP kinases [42, 45, 46], leading to metabolic dysregulations [42] such as inhibitions of glycogen synthesis, impaired translocation of Glut4 to plasmatic membrane, or antilipolytic effects of insulin in white adipose tissue [39, 46].

Meta-analyses from 448 articles reporting shift work and health consequence have detected a strong relationship between shift work, a potent chronodisruptor, and diabetes mellitus type 2 (odd ratio about 1.42 times higher than day workers) [47]. However, this is not the only chronodisruption which we are exposed too. In animal models exposed to light/dark patterns similar to shift work, the length of the working day and changes of feeding times induce a modification of the liver weight and plasmatic glucose and a modification of the circadian profile for glucose, insulin, and triglyceride [48]. These findings add to the observation in the circadian disruption in mutant animals, showing alterations of cholesterol metabolism, abolition of the circadian production of glycerol, free fatty acids, and impaired expression of rate-limiting lipolytic enzymes such as lipase [49], all of which add to increased weight gain, adipocyte hypertrophy [49], high level of glucose, glucose intolerance, and hypersecretion of insulin [49–52]. Polymorphic variations of clock genes can be incrementing the risks of developing a disease similar to chronodisruption by environmental changes as it has been detected in human metabolic disorders (see Table 1). For example, the genotyping of clock genes in 346 Greek pregnant women and their risk of diabetes was performed, detecting that the polymorphisms of* Bmal1 rs7950226* and* rs11022775* are associated with gestational diabetes mellitus (*P* = 0.025, OR = 1.46 and *P* = 4.455*e* − 06, OR = 2.64, resp.), while the study of the haplotype analysis of* rs7950226/rs11022775* showed a major frequency in women with gestational diabetes mellitus (*P* = 0.0069, OR = 6.96) [33]. Similarly, a study performed in subjects from the United Kingdom and Pakistan reported that* Cry1* and* Cry2* polymorphisms* rs2292912* and* rs12315175*, respectively, are associated with diabetes (*P* = 0.015 and 0.008, resp.). Moreover, the variant* rs12315175* for* Cry2* has a tendency to elevate the risk at about 5% compared to the variation of* Cry1* (OR = 1.05 and 0.95, resp.) [53]. Also, the authors did not find associations between diabetes and* Bmal1* polymorphisms* rs7950226* and* rs11022775* [53], as occurs in gestational diabetes mellitus [33]. Genotyping of 19,000 adults from Northern Sweden for variants rs8192440 for Cry1 and rs11605924 for Cry2 is associated with higher level of glucose concentrations at 2 hours (*P* = 0.06 and *P* = 0.005, resp.) [54].

Another study genotyping 1304 individuals from 424 British families, containing at least one patient with diabetes type 2 (diabetes in families study collection), demonstrates the relation between* Bmal1* polymorphisms* rs7950226* and* rs11022775* and diabetes (*P* = 0.002), reinforcing what was previously described [55]. Similar relations are observed for Bmal2 polymorphism* rs7958822* in obese men and women (OR 2.2 and 2.7, resp.) [56] and the deletion/insertion of 54 base pair sequences of five repeat alleles on* Per3* gene (*rs57875989*) [57, 58]. In contrast, the* Per2* polymorphism* rs7602358* is associated with a protection from type 2 diabetes in the UK population, which suggests that not all polymorphisms are negative for health [53].

At the level of dyslipidemia, an interesting correlation has been detected between small dense LDL level and polymorphism of clock genes. In individuals carrying the polymorphism associated with the* Clock* gene (*rs1801260*), the genotype TT or TC showed a major level of small dense LDL, which leads to increased triglycerides and increased risk of cardiovascular and obesity diseases [59], similar to the “metabolic syndrome” [39]. Likewise, genotyping in the Japanese population identified* Clock* gene polymorphism* rs1801260* associated with higher odds ratio (OR; 1.5) of type 2 diabetes [60]; in contrast, a multicenter study detected the* Clock* polymorphism rs4580704 is associated with prevention of diabetes and cardiovascular disease [61]. The haplotypes of* rs10002541* and* rs4864546* from* Clock* (CG and TG variations) are associated with abdominal obesity in Chinese population (OR 0.74 and 1.70, resp.) [62] and polymorphism* rs4864546 is associated with* low level of HDL/apolipoprotein A1 ratio in Spain [63]. Moreover, Clock polymorphisms* rs12649507* and* rs3749474* are associated with higher intake of polyunsaturated fatty acid [64] and fat intake [65], which can modify the body mass index (BMI). This is an antecedent which, bearing in mind the ideas above, suggests the genetic components of the circadian system as a critical factor in the development of metabolic diseases.

## 3. Clock Genes and Infertility

In humans, infertility affects about 9% of couples, and about one-third of infertility cases associated with idiopathic male infertility are multifactorial, with 50% due to genetic abnormalities [88]. The circadian system is important during reproduction and development [89, 90]; for example, it is involved in the timing of the LH surge [90, 91], stimulation of ovulation [90, 92], and regulating the level of steroidogenic acute regulatory protein (StAR) expression and is critical for cholesterol translocation inside mitochondria and sperm count [93].

The genetic factors of clock genes are implicated in the pathogenesis of infertility (see Table 2), as it occurs in male partners of infertile couples. The genotyping of male partners detected a correlation between single nucleotide polymorphism of* Clock rs11932595*,* rs6811520*, and* 6850524*, with infertility (*P* < 0.05 and OR ranged between 1.4 and 1.9). Similarly, the analysis of* Bmal1* polymorphism* rs4757144* in Slovenian and Serbian Caucasian men showed a significant correlation with infertility (*P* = 0.047) [31], suggesting that clock genes Clock and Bmal1 contribute to successful fertilization.

## 4. Clock Genes, Mood Disorder, and Others

Mood or affective disorders are important causes of morbidity, where we can highlight depressive pathologies and hypomaniac and maniac disorders as major diseases affecting the population [29, 66], and it can be highlighted that depressive pathologies are the third largest source of morbidity in the world [29]. Curiously, a potent correlation has been detected between mood disorders, sleep/activity, and the circadian system [94] suggesting that the circadian system can be modulating the neuronal activity [95]. During pregnancy, the prevalence of mental illnesses in women is about 8% [96], suggesting an imbalance between physiology of sleep and/or the circadian system. This can be observed in the onset of a mental disease as has been observed in the impaired circadian production of melatonin during the pregnancy of depressed women (approximately ≤34 weeks of gestation), which shows an advance onset time of melatonin production of about 40 minutes and a minor production during the dark hours [97, 98]. Moreover, workers who do shift work (circadian disruption) showed decreased alertness, cognitive functions, mood, social and work activities, and health [26, 99], which are associated with an impaired circadian system via melatonin suppression, all of which leads to the appearance of a mood disorder [99]. For this reason, we can say that the circadian system may contribute to the risk of developing a mood disorder and the genetic component of clock genes can be involved in the development of mood pathologies [94] (see Table 2).

At the genetic component level of the circadian system, a study of 744 people who carry polymorphisms* rs2291739* and* rs11171856* from TIM, a member of the clock gene family which interacts with Per1-2 proteins [100, 101], showed that they have an elevated risk of developing mood disorders ranging between 19 and 23% (OR 1.19 and 1.23, resp.) [66]. Moreover, gene variants of positive regulator Clock have also been associated with mood disorders. People who carry the variants* rs1801260* and* rs11932595* showed a major risk of developing a bipolar disorder ranging between 45 and 37%, respectively, in comparison to a patients without polymorphism (OR 1.45 and 1.37, resp.). In the same study, it was also observed that the polymorphisms* rs2291739* and* rs11171856* from TIM gene are associated with unipolar disease, with a risk ranging between 37 and 40% in comparison to a patient without polymorphism (OR 1.37 and 1.40, resp.). Similarly,* Bmal1* polymorphisms* rs1160996C/rs11022779G/rs1122780T* (haplotype) are associated with mood disorders [66] and bipolar disease [29, 66]. It has also been seen that the bipolar pathologies are associated with other genetic variations of* Bmal1* gene (*rs4757144*,* rs1982350*, and* rs1481892*) and the negative regulator* Per3* (*rs2859387*) [69]. Curiously, a study performed an Indian families reported the* Bmal1* polymorphisms rs2279287 are associated with seasonal affective disorder [70].

In addition, the gene variant of* Cry2* gene (rs4132063) [71] and deletion/insertion of 54 base pair sequences on* Per3* gene (*rs57875989*) [67] may also be contributing to mood disorders via increased risk of developing bipolar disease. For example, a study in South India showed the prevalence of five repeat homozygotes from* rs57875989* is associated with bipolar disease (OR 1.72) but not with schizophrenia [68].

At the level of depressive pathologies, a study performed in China compared 485 subjects (control) to 105 patients suffering from a depression disorder. The genotyping for clock genes showed that the variants of* Cry1* (*rs2287161*) and* Cry2* (rs10838524) are correlated to the depression disease with an odds ratio of 1.75 (*P* = 0.012), which suggests that a patient with the allele has 1.75 times more risk of developing depression [30]. Moreover the authors detected a single nucleotide polymorphism for TEF gene, the variant* rs738499*, is associated with 2.22 times more risk of development of depression (odds ratio of 2.22. *P* < 0.001) than people not carrying the TEF polymorphism [30].

Finally, in a study performed in young adults, the risk of excessive intake of alcohol is minor when the polymorphism of* Per2* gene rs56013859 is present, suggesting a possible protector role for allelic variation of clock genes in addictions [34]. In fact, this suggests that some polymorphisms are not negative for our health and it is necessary to study them in greater depth.

## 5. Clock Genes and Cancer

Cellular proliferation is a critical event for the survival and restitution of tissues of all living things and the circadian system is capable of delivering temporary information to the cell cycle [102–104]. However, an uncontrolled cellular proliferation and an excessive tissue growth are observed by an altered cellular cycle during cancer [105], a pathology that showed a higher incidence in patients exposed to an impaired circadian system such as a shiftwork [106, 107]. The cell cycle is a finely regulated process from a cell that is capable of generating multiple cells through a series of cell divisions [101], including four critical and successive steps named G1 phase (growth phase 1), S phase (synthesis), G2 (growth phase 2), and M phase (mitosis) [108]. During the cell cycle, cyclin-dependent kinases (CDK) are critical for the transition between different stages, for example, cdc2 plus cyclin B protein kinase form the complex CDK1 which regulates G2/M transition [108, 109]. During DNA replication, there are a number of controls or checkpoints that are critical in the cell cycle when DNA damage is detected: the activation of the Rad protein, which induces the action of CDs 1 proteins, is triggered, as well as Chk1 which are involved in cell cycle arrest via action of Wee-1 and Mik-1 proteins [109].

Partial hepatectomy induces hepatocytes to enter the cell division for liver regeneration. However, the efficiency of this process changes depending on the time of the day when the lesion occurred. A lesion in the liver, occurring during the last light hours, induced a massive entry to M phase compared to a lesion which occurs early in the morning, which shows that the hour of surgery and the circadian system are critical for liver regeneration [20]. Similarly, oral mucosa is a highly proliferative tissue and it showed a circadian expression of clock genes* Bmal1*,* Per1,* and* Cry1* and thymidylate synthase activity, critical for DNA synthesis during phase S. Curiously, the peak of* Per1* mRNA precedes the peak of thymidylate synthase activity [110], which suggests that the circadian system modulates the cellular proliferation in mucosa.

Wee-1 gene regulates cellular proliferation, and its promoter has three conserved sequence CACGTG (E-box) critical for the circadian expression of Wee-1 [20]. Its protein is capable of inactivating the cdc2-cyclin B complex via phosphorylation which inhibits transition between G2/M. Curiously, knock-down of* Bmal1* in carcinoma cells of the colon (c26 cells), fibroblast cells (L929 cell), and intestine epithelial cells (IECs) produces cellular proliferation* in vitro* and increments the size of tumor cells injected subcutaneously, via the inhibition of apoptosis and the reduction of the time transition between G2/M [102], reduction of p53 and Wee-1 expression [103]. Moreover, the knock-out for clock genes* Bmal1* y* Per2* in mice previously exposed to gamma radiation caused in mice hyperplasic growth and development of lymphoma, hepatic carcinomas, ovary tumors, and osteosarcomas via reduction of p53 expression [103]. A precedent that reinforces the idea that clock genes can be modulators of cellular cycle via Wee-1 and p53 proteins.

At the genetic component level, different studies reveal the importance of the polymorphic variant of clock genes (see Table 3) and how the Chrono-type modulates the risk of cancer, such as observed in breast cancer, in which the risk is more elevated in premenopausal women (OR, 2.43 and 2.55; resp.) [77]. Similarly, women showed evening or night preference such as shown in the case of a study performed on Norwegian nurses working in night shift which revealed two polymorphisms associated with breast cancer. The risk is incremented when women spend more time working during night hours, but this risk is higher when women have the alleles for* Bmal1 rs2290035* (OR 1.91),* rs969485* (OR 1.64), and* rs3903529* (OR 2.77) or variant* rs3750420* (OR 1.6) of* Roreb* gene [84]. Similarly, an incremented risk is associated with breast cancer in women from France when they have the allele* rs11932595* in* Clock* gene (OR = 0.74) or in Connecticut (USA) when they have the polymorphisms* rs7698022* (OR 1.34) and* rs1048004* (OR, 1.43) [73]. Curiously, when Norwegian nurses are exposed to shift work during four nights, they showed three times more risk (OR 2.75) of developing breast cancer when they were carrying Clock polymorphism* rs11133373*, which suggests that the disruption of endogenous circadian rhythm by polymorphism associated with Clock genes increases the risk of cancer [84]. Moreover, Clock gene expression is induced in breast tissue from patients with breast cancer; this expression is associated with hypomethylation of* Clock* gene. The silencing of* Clock* gene lowered when women carry polymorphisms associated with cancer such as rs10448004 and rs7698022, which suggests that* Clock* gene is a critical protagonist of cancer development [73].

At level of colorectal cancer, a strong correlation is detected between cancer and the genetic component of the circadian system. For example, a polymorphism in the* Clock *gene (*rs1801260*) showed a major prevalence in a cancer patient (*P* < 0.0001, OR = 1.78 for C-allele) compared to a control patient [32]. Similarly, a screening performed in South Carolina, USA, showed that a variation by deletion/insertion of a 54 base pair sequence on Per3 gene (*rs57875989*) is associated with a higher risk of colorectal adenoma formation with odds ratio within 2.1–5.1 [78]. Moreover, a population study performed on the residents of King County, Washington (USA), detected six different types of polymorphism, which are significantly associated with the risk and aggressiveness of prostate cancer. These genetics variants are* rs1012477* for Per3 (OR 1.3),* rs7602358* for Per2 (OR 1.24), and* rs2289591* in Per1 (OR 1.7) [76]. Similarly, a genotyping of a patient with prostate cancer showed a strong correlation between Cry1 polymorphisms rs7297614, rs1921126, and rs12315175 and fatal prostate cancer (OR mean within 1.5–2) [85] and a study conducted in China showed that the Cry2 variant rs1401417 and the deletion/insertion of 54 base pair sequences on Per3 gene (*rs57875989*) were associated with a major risk of developing prostate cancer (OR; 1.7 and 1.3, resp.) [79].

A study performed in Brazilian patients with pulmonary cancer showed a strong correlation of Per3 polymorphism* rs228644* and the risk of cancer (OR 1.99). Moreover, the authors reported the ancestral haplotypes for Per3* rs228729*,* rs228727*,* rs707467*,* rs228644,* and rs10462020 are associated with a higher cancer frequency [83]; similarly, a meta-analysis performed by literature search showed that the variant insertion/deletion of Per3 rs57875989 is associated with an increase of cancer susceptibility in about 17% [80] or 70% [81]. In a similar way, a soft risk is associated with breast cancer in premenopausal women from India [77], which suggests there is a relation between Per3 and cellular proliferation.

The frequency analysis of 1,538 breast cancer cases and 1,605 controls in China for clock gene variants showed the strong associations between three SNPs in circadian clock genes and the risk of developing breast cancer. The variants for Cry1* rs1056560* are correlated to cancer, which elevate the risk in about 11% (OR 1.11); Per2 rs934945 in about 15% (OR 1.15); and Clock rs3805151 in about 35% (OR 1.35) [74]. In contrast, TIM protection for breast cancer development is detected in patients that carry the C-allele of* rs7302060* (OR, 0.54). The G allele of rs2291738 and the C-allele of rs7302060 are associated with reduced risk of breast cancer among estrogen receptor (−) or progesterone receptor (−) positive breast cancer cases (OR, 0.46 and 0.36, resp.) [87].

The exogenous expression of human clock in cell lines of colorectal carcinoma induces the cellular proliferation in about 28%. In contrast, knock-down of endogenous Clock gene expression inhibits the cell proliferation in about 34% [111]. Moreover, exogenous Clock inhibits the apoptosis (42% reduction) via inhibition on apoptosis associated proteins expression of Bax and Bid and the increase of phosphorylation of Akt [111].* In vivo* experiments by xenograft transplant of colorectal carcinoma cell line transduced with clock increase the tumor volume and tumor weight in about 61% and a 91%, respectively [111]. However, the pharmacological inhibition of Cry in human breast cancer by treatment with pharmacological agent KS15 [112] inhibits the proliferation and cell viability by stimulation of Wee-1 expression [112] and stimulates the activity of heterodimer complex Bmal1:Clock [112, 113].

Curiously, two polymorphisms for clock gene Cry2, rs11038689 and rs1401417, have a protective action over the mammary cancer [84], which suggests that not all polymorphisms associated with clock genes are negative for our health.

Non-Hodgkin's lymphoma is characterized by lymphoproliferation and advanced clinical stages, invasion to other tissues, and death. The estimated deaths from non-Hodgkin's lymphoma in the United States amounted to 19,790 during 2015 [114]. Analysis of Cry2 variants showed the polymorphisms* rs11038689*,* rs7123390*, and* rs1401417* increase the risk of lymphoma (*OR, 2.34; 2.40 and 2.97, resp.*) [86]. Moreover, a minor association of clock gene variants and glioma is detected for* Per1 rs2585405, Clock rs11133391,* and* Cry1 rs12315175* (*OR, 1.16; 1.08 and 1.02, resp*.) [75].

Genotyping of Han Chinese patients diagnosed with primary hepatocellular carcinoma showed an association between single SNPs of Per3* rs228669* and Cry1* rs3809236* with odds ratio of 1.41 and 1.26, respectively [82]. These precedents reinforce the idea that clock genes can be modulators of cellular cycle and that modulates the risk of cancer.

## 6. Conclusion

The circadian system is a supraphysiological system which modulates different physiological systems, and any alteration of this can have a negative impact on human health. Different chronodisruptors have been described in literature such as light/dark pattern and inhibition of melatonin production as occurs in shifts work. However, other factors can be contributing on a minor scale, such as mealtimes or a genetic component.

The relevance of the genetic variation of clock genes and how it can interact with the environment is unknown. But it has been described that the genetic component in the population predisposes the development of different pathologies such as diabetes, dyslipidemias, obesity, mood disorders, and addiction, all of which suggest the importance of this system to our health. However, it is also necessary to say that the genetic component could be protecting our health such as in the case of polymorphisms associated with Per2 and Cry2 genes in diabetes, alcohol intake, and cancer.

These findings will need to be implemented and evaluated at the genetic interaction level and also the way in which the environment factors trigger the expression of these pathologies will be examined. Finally, prospective studies are necessary to assess the predictive potential of these markers and to implement early treatment with consequent cost reduction for the health system.

## Figures and Tables

**Table 1:** Polymorphisms of clock genes associated with metabolic disorders.

Gene	SNPs	Variation	Localization	Pathology	Reference
*Clock*	*rs1801260*	T>C	Chromosome 4; 3′ UTR; *Homo sapiens*	Dyslipidemia and diabetes	[59, 60]
	*rs10002541*	T>C	Chromosome 4; intron; *Homo sapiens*	Diabetes	[62]
	*rs12649507*	G>A	Chromosome 4; intron; *Homo sapiens*	Food intake	[64]
	*rs4580704*	G>C	Chromosome 4; intron; *Homo sapiens*	Diabetes, cardiovascular disease, and HDL level	[61, 63]
	*rs3749474*	C>T	Chromosome 4; 3-UTR; *Homo sapiens*	Food intake	[65]
	*rs4864546*	G>A	Chromosome 4; near gene-5′; *Homo sapiens*.	Diabetes	[62]

*Per2*	*rs7602358*	G>T	Chromosome 2; intron; *Homo sapiens*	Diabetes	[32]

*Per3*	*rs57875989 *	Deletion/insertion	Chromosome 1; 5′ near gene; *Homo sapiens*	Diabetes	[57, 58]

*Bmal1*	*rs11022775*	C>T	Chromosome 11; intron; *Homo sapiens*	Gestational diabetes mellitus	[33]
	*rs7950226*	G>A	Chromosome 11; intron; *Homo sapiens*	Gestational diabetes mellitus	[33]

*Bmal2*	*rs7958822*	G>A	Chromosome 12; intron; *Homo sapiens*	Diabetes	[56]

*Cry1*	*rs12315175*	T>C	Chromosome 12; *Homo sapiens*	Diabetes	[53]
	*rs8192440*	A>G	Chromosome 12; CDS; *Homo sapiens*	Diabetes	[54]

*Cry2*	*rs11605924*	A>C	Chromosome 11; intron; *Homo sapiens*	Diabetes	[54]
	*rs2292912*	C>G/T	Chromosome 11; intron; *Homo sapiens*	Diabetes	[53]

**Table 2:** Polymorphisms of clock genes associated with infertility and mood disorders.

Gene	SNPs	Variation	Localization	Pathology	Reference
*Clock*	*rs1801260*	T>C	Chromosome 4; 3′ UTR; *Homo sapiens*	Bipolar disease	[66]
	*rs11932595*	A>G	Chromosome 4; intron; *Homo sapiens*	Infertility and bipolar disease	[31, 66]
	*rs6811520*	T>C	Chromosome 4; intron; *Homo sapiens*	Infertility	[31]
	*rs6850524*	G>C	Chromosome 4; intron; *Homo sapiens*	Infertility	[31]

*Per2*	*rs56013859*	T>C	Chromosome 2; intron; *Homo sapiens*	Alcohol intake	[34]

*Per3*	*rs57875989 *	Deletion/insertion	Chromosome 1; 5′ near gene; *Homo sapiens*	Bipolar disease	[67, 68]
	*rs2859387*	G>A/C	Chromosome 1; 5′ near gene; *Homo sapiens*	Bipolar disease	[69]

*Bmal1*	*rs4757144*	G>A	Chromosome 11; intron; *Homo sapiens*	Infertility and bipolar disease	[31, 69]
	*rs1481892*	G>C	Chromosome 11; intron; *Homo sapiens*	Bipolar disease	[69]
	*rs1982350*	A>G	Chromosome 11; intron; *Homo sapiens*	Bipolar disease	[69]
	*rs2279287*	T>C	Chromosome 11; near-Gene 5′; *Homo sapiens*	Seasonal affective disorder	[70]

*Cry1*	*rs2287161*	G>C	Chromosome 12; upstream variant 2 KB; *Homo sapiens*	Depression disease	[30]

*Cry2*	*rs4132063*	C>T	Chromosome 11; *Homo sapiens*	Depression disease	[71]
	*rs10838524*	A>G	Chromosome 11; *Homo sapiens*	Depression disease	[71]

*Tef*	*rs738499 *	G>T	Chromosome 22; intron upstream variant 2 KB; *Homo sapiens*	Depression disease	[30]

*TIM*	*rs2291739*	G>A/C	Chromosome 12; exon; *Homo sapiens*	Unipolar disease	[66]
	*rs11171856*	C>T	Chromosome 12; intron; *Homo sapiens*	Unipolar disease	[66]

**Table 3:** Polymorphisms of clock genes associated with cancer.

Gene	SNPs	Variation	Localization	Pathology	Reference
*Clock*	*rs1801260*	T>C	Chromosome 4; 3′ UTR; *Homo sapiens*	Colorectal cancer	[32]
	*rs11932595*	A>G	Chromosome 4; intron; *Homo sapiens*	Breast cancer	[72]
	*rs7698022*	C>A	Chromosome 4; intron; *Homo sapiens*	Breast cancer	[73]
	*rs11932595*	A>G	Chromosome 4; intron; *Homo sapiens*	Breast cancer	[73]
	*rs1048004*	G>T	Chromosome 4; 3′ UTR; *Homo sapiens*	Breast cancer	[73]
	*rs3805151*	C>T	Chromosome 4; intron-6; *Homo sapiens*	Breast cancer	[74]
	*rs11133391*	T>C	Chromosome 4; intron; *Homo sapiens*	Glioma	[75]

*Per1*	*rs2585405*	C>G	Chromosome 17; missense; *Homo sapiens*	Glioma	[75]
	*rs2289591*	G>T	Chromosome 17; 5′ near gene; *Homo sapiens*	Prostate cancer	[76]

*Per2*	*rs7602358*	G>T	Chromosome 2; intron; *Homo sapiens*	Prostate cancer	[76]
	*rs934945: *	G>A	Chromosome 2; Exon-23; *Homo sapiens*	Breast cancer	[74]

*Per3*	*rs1012477*	G>C	Chromosome 1; 5′ near gene; *Homo sapiens*	Prostate cancer	[76]
	*rs57875989 *	Deletion/insertion	Chromosome 1; 5′ near gene; *Homo sapiens*	Colorectal cancer, breast cancer Prostate cancer	[74, 77–81]
	*rs228669*	T>C	Chromosome 1; CDS; *Homo sapiens*	Hepatocellular carcinoma	[82]
	*rs228644*	G>A	Chromosome 1; intron; *Homo sapiens*	Lung cancer	[83]

*Bmal1*	*rs2290035*	T>A	Chromosome 11; intron; *Homo sapiens*	Breast cancer	[84]
	*rs969485*	G>A	Chromosome 11; intron; *Homo sapiens*	Breast cancer	[84]

*Cry1*	*rs3809236*	C>T	Chromosome 12; 5′ UTR *Homo sapiens*	Hepatocellular carcinoma	[82]
	*rs1056560*	T>G	Chromosome 12; exon-13 *Homo sapiens*	Breast cancer	[74]
	*rs7297614*	C>T	Chromosome 12; 5′ UTR *Homo sapiens*	Prostate cancer	[85]
	*rs1921126*	C>T	Chromosome 12; intron *Homo sapiens*	Prostate cancer	[85]
	*rs12315175*	T>C	Chromosome 12; 5′ UTR *Homo sapiens*	Prostate cancer	[85]
	*rs12315175*	T>C	Chromosome 12; noncoding sequence	Glioma	[75]

*Cry2*	*rs11038689 *	A>G	Chromosome 11; intron; *Homo sapiens*	Breast cancer	[84]
	*rs1401417*	C>G	Chromosome 11; intron; *Homo sapiens*	Breast cancer Prostate cancer	[79, 84]
	*rs11038689*	A>G	Chromosome 11; intron; *Homo sapiens*	Non-Hodgkin's lymphoma	[86]
	*rs7123390*	G>A	Chromosome 11; intron; *Homo sapiens*	Non-Hodgkin's lymphoma	[86]
	*rs1401417*	C>G	Chromosome 11; intron; *Homo sapiens*	Non-Hodgkin's lymphoma	[86]

*TIM*	*rs7302060*	T>C	Chromosome 12; intron; *Homo sapiens*	Breast cancer	[87]
	*rs2291738*	T>C	Chromosome 12; intron; *Homo sapiens*	Breast cancer	[87]

*ROR-b*	*rs3750420*	C>T	Chromosome 9; intron; *Homo sapiens*	Breast cancer	[84]
	*rs3903529*	T>A	Chromosome 9; intron; *Homo sapiens*	Breast cancer	[84]

## Glossary

Bmal1: Aryl hydrocarbon receptor nuclear translocator-like protein 1
Clock: Circadian locomotor output cycles kaput
Per: Homolog of period, drosophila
E-box: Promoter sequence for binding of clock-Bmal1 complex (CACGTG)
DBP: Albumin D-site-binding protein
VEGF: Vascular endothelial growth factor
StAR: Steroidogenic acute regulatory protein
3*β*-HSD: 3-Beta-hydroxysteroid dehydrogenase.
